# Deletion of voltage-gated calcium channels in astrocytes decreases neuroinflammation and demyelination in a murine model of multiple sclerosis

**DOI:** 10.1186/s12974-023-02948-x

**Published:** 2023-11-14

**Authors:** G. E. Denaroso, Z. Smith, C. G. Angeliu, V. T. Cheli, C. Wang, P. M. Paez

**Affiliations:** Institute for Myelin and Glia Exploration, Department of Pharmacology and Toxicology, Jacobs School of Medicine and Biomedical Sciences, The State University of New York, University at Buffalo, NYS Center of Excellence, 701 Ellicott St., Buffalo, NY 14203 USA

**Keywords:** Myelin, Astrocytes, Astrogliosis, Voltage-gated calcium channels, Cav1.2 channels, Demyelinating diseases, EAE, Multiple sclerosis

## Abstract

The experimental autoimmune encephalomyelitis (EAE) model of multiple sclerosis was used in combination with a Cav1.2 conditional knock-out mouse (Cav1.2^KO^) to study the role of astrocytic voltage-gated Ca^++^ channels in autoimmune CNS inflammation and demyelination. Cav1.2 channels were specifically ablated in Glast-1-positive astrocytes by means of the *Cre-lox* system before EAE induction. After immunization, motor activity was assessed daily, and a clinical score was given based on the severity of EAE symptoms. Cav1.2 deletion in astrocytes significantly reduced the severity of the disease. While no changes were found in the day of onset and peak disease severity, EAE mean clinical score was lower in Cav1.2^KO^ animals during the chronic phase of the disease. This corresponded to better performance on the rotarod and increased motor activity in Cav1.2^KO^ mice. Furthermore, decreased numbers of reactive astrocytes, activated microglia, and infiltrating lymphocytes were found in the lumbar section of the spinal cord of Cav1.2^KO^ mice 40 days after immunization. The degree of myelin protein loss and size of demyelinated lesions were also attenuated in Cav1.2^KO^ spinal cords. Similar results were found in EAE animals treated with nimodipine, a Cav1.2 Ca^++^ channel inhibitor with high affinity to the CNS. Mice injected with nimodipine during the acute and chronic phases of the disease exhibited lower numbers of reactive astrocytes, activated microglial, and infiltrating immune cells, as well as fewer demyelinated lesions in the spinal cord. These changes were correlated with improved clinical scores and motor performance. In summary, these data suggest that antagonizing Cav1.2 channels in astrocytes during EAE alleviates neuroinflammation and protects the spinal cord from autoimmune demyelination.

## Introduction

Following neurological injury or disease, astrocytes undergo several functional and morphological changes to minimize CNS damage and expedite repair in a process called astrogliosis [29]. However, the loss of normal astrocytic functions and acquisition of abnormal functions during astrogliosis can be deleterious for CNS recovery [16, 30]. Control of intracellular Ca^++^ levels is essential for several homeostatic functions, as well as reactive responses, exhibited by astrocytes [10, 28]. Voltage-gated Ca^++^ channels (VGCCs) are the primary mechanism allowing for Ca^++^ entry into most excitable cells [31]. Although astrocytes are not excitable, these cells express several voltage-dependent ion channels, including VGCCs [20]. Specifically, the L-type Cav1.2 α isoform is highly expressed by astrocytes and upregulated during astrogliosis [5]. Importantly, knocking out Cav1.2 in astrocytes attenuated Ca^++^ influx post-membrane depolarization and inhibited astrocyte activation and proliferation following administration of the endotoxin lipopolysaccharide (LPS) and mechanical insult [5]. L-type channel antagonists also reduced the density of reactive astrocytes, astroglia hypertrophy and proliferation, and the release of pro-inflammatory cytokines [5]. Accordingly, we have shown that ablating astrocytic Cav1.2 channels in vivo decreased the activation and proliferation of astrocytes and microglial cells, enhanced myelin regeneration, and reduced cortical and white matter levels of pro-inflammatory factors in the cuprizone model of demyelination [38].

Reactive astrogliosis is especially damaging in demyelinating diseases, such as multiple sclerosis (MS) [14]. MS is an autoimmune disorder in which peripheral immune cells infiltrate the brain and spinal cord and produce focal demyelinated lesions. Experimental autoimmune encephalomyelitis (EAE) is a widely used animal model for MS, and has been shown to closely emulate the pathophysiological sequence of the human disease [6]. Utilizing this model, investigators have demonstrated that MS pathogenesis not only requires inflammation by peripheral immune cells, but also the activation and proliferation of CNS resident cells, such as astrocytes and microglia [7, 22]. Although it has been shown that astrocytic Cav1.2 VGCCs are upregulated during astrogliosis and crucial to the induction and proliferation of reactive astrocytes in a toxin-induced myelin degeneration context, the role of these Ca^++^ channels in autoimmune-related demyelination is unknown. Therefore, we tested whether eliminating or inhibiting astrocytic Cav1.2 channels would attenuate astrogliosis, neuroinflammation, and demyelination in the EAE model of MS. We found that reducing voltage-gated Ca^++^ influx in astrocytes significantly attenuated neuroinflammation and myelin degeneration in EAE animals. The specific ablation of Cav1.2 channels in astrocytes reduced myelin loss and EAE symptoms during the chronic phase of the disease and, congruently, nimodipine treatment ameliorated EAE severity and protected the myelin of the spinal cord. Collectively, these findings suggest that Cav1.2 channels in astrocytes are potential therapeutic targets to lessen CNS inflammation and myelin loss in autoimmune demyelinating diseases.

## Materials and methods

### Transgenic mice

All animal subjects resided in the vivarium of the University at Buffalo (UB) Division of Laboratory Animal Medicine. Procedures were granted approval by UB’s Animal Care and Use Committee and conducted in accordance with the National Institutes of Health’s *Guide for the Care and Use of Laboratory Animals*. Murphy Geoffrey (University of Michigan, Ann Arbor) provided the heterozygous *floxed* Cav1.2 mouse line, while The Jackson Laboratory supplied the hemizygous Glast1-*Cre*ER^T2^ transgenic line (Jackson Mice, #012586). Experimental transgenic mice were generated by crossing the Cav1.2 line with the Glast1-*Cre*ER^T2^ transgenic line. For simplicity, conditional knockout mice (Cav1.2^f/f^, Glast1*Cre*^+/−^) and *floxed* controls (Cav1.2^f/f^, Glast1*Cre*^−/−^) will be referred to as Cav1.2^KO^ and controls, respectively, for the remainder of this manuscript. Female mice were used exclusively for all experiments described in this text.

### Experimental autoimmune encephalomyelitis (EAE)

Postnatal day (P)60 C57BL/6, conditional knockout (Cav1.2^f/f^, Glast1*Cre*^+/−^) and *floxed* controls (Cav1.2^f/f^, Glast1*Cre*^−/−^) mice were immunized using the EAE induction kit by Hooke Labs (Hooke Laboratories; CN: EK2110). EAE was induced via subcutaneous injection with 200 μg of myelin oligodendrocyte glycoprotein 35–55 (MOG_35–55_) in emulsion with complete Freund's adjuvant (CFA). On post-immunization day (D)0 and D1, pertussis toxin (PTX) (110 ng per mouse) in PBS was administered intraperitoneally. Animals were monitored daily for disease symptoms from D7 to D39. Disease severity was quantified on a numerical scale from 0 to 5 as outlined by Hooke Laboratories: 0, no disease symptoms; 0.5, lack of tension in tip of tail; 1, entire tail is limp; 1.5, limp tail and hind limb inhibition; 2, limp tail and wobbly walk; 2.5, limp tail and dragging of hind feet; 3, limp tail and complete hind limb paralysis; 3.5, fails backflip test and paralyzed hind limbs are held together on one side of body; 4, limp tail and complete hind limb paralysis with partial front limb paralysis; 4.5, immobile and largely unresponsive to contact; 5, moribund.

### Mice treatments

To induce *Cre* activity in Glast1-positive astrocytes conditional knockout mice (Cav1.2^f/f^, Glast1*Cre*^+/−^) and *floxed* controls (Cav1.2^f/f^, Glast1*Cre*^−/−^) were injected daily with tamoxifen (Sigma-Aldrich; CN: T5648) (100 mg/kg) for 5 days, for a total of five injections, beginning 2 weeks prior to EAE induction. Tamoxifen (Sigma-Aldrich) was dissolved and sonicated in autoclaved corn oil for stock solutions (20 mg/ml) and injected intraperitoneally. For the pharmacological experiments, either nimodipine (Sigma-Aldrich; CN: N149) (10 mg/kg) mixed with vehicle solution [5% ethanol, 5% DMSO (Sigma-Aldrich), 40% PEG 400 (Sigma-Aldrich) and normal saline], or exclusively the vehicle solution, was administered subcutaneously to C57BL/6 female mice every other day for 10 days, for a total of six injections during the acute (D7–17) or peak phase (D14–24) of the disease. Since nimodipine is highly photosensitive, all procedures, including stock preparation and administration, were performed under light-protected conditions.

### Behavioral assays

One week prior to immunization, all animals underwent 2 days of training for the rotarod performance test, set to an acceleration protocol of 4–40 rpm over 300 s. Coordinated motor activity was tested daily with the same settings from D7–D39. Latency to fall off the rotarod apparatus was recorded for each subject in seconds. Motor activity was also assessed via the quantification of vertical and horizontal movements. Pitch counters were used to track how often subjects either crossed the midline of their cage or stood up on their hind legs over the course of 5 min. All animals were weighed on a digital gram scale every other day starting 1 week after EAE induction.

### qPCR

Total RNA was extracted and purified from the lumbar section of spinal cords using Trizol (Invitrogen; CN:15596018). RNA content was estimated by measuring the absorbance at 260 nm and the purity was assessed by measuring the ratio of absorbance: 260/280 nm. Briefly, 500 µg of RNA was reverse transcribed using Prime Script™ 1st strand cDNA Synthesis Kit (Takara; CN: 6110A). qPCR was performed using a CFX96 Touch Real-Time PCR Detection Systems (Bio-Rad) and SYBR™ Green PCR Master Mix (Applied Biosystems; CN: 4309155). qPCR conditions were as follows: polymerase activation 95 °C, 10 min, followed by 40 cycles; denaturation at 95 °C, 15 s; primer annealing/elongation at 60 °C, 60 s. Primer specificity was analyzed adding a melting curve for each reaction. Quantification of PCR products was conducted using the Pfaffl method. Quantities of mRNA were normalized to the housekeeping genes GAPDH and TBP.

### Western blot

Total proteins were extracted as described in Santiago González et al. [25]. Proteins (20 μg/line) were separated with NuPAGE® Novex® 4–12% Bis–Tris Protein Gels (Invitrogen; CN: NP0321) and electroblotted onto PDVF membranes. Membranes were blocked overnight at 4 °C with 5% non-fat milk, 0.1% tween-20 in PBS. Primary antibodies were diluted in a blocking solution and membranes incubated for 3 h at room temperature with agitation. Protein bands were detected by chemiluminescence using the Amersham ECL kit (GE Healthcare; CN: RPN2135) with horseradish peroxidase-conjugated secondary antibodies (GE Healthcare) and scanned with a C-Digit Bot Scanner (LI-COR). Protein bands were quantified using the Image Studio™ Software (LI-COR). Primary antibodies were against the following antigens: β-actin (mouse; 1:10,000; Sigma-Aldrich; CN: A5441), CD68 (mouse; 1:1000; Biolegend; CN: 137002), CNP (mouse; 1:1000; Neo-Markers; CN: MS349P), GFAP (rabbit; 1:20,000; Dako; CN: Z0334), Iba1 (rabbit; 1:2000; Wako; CN: 01919741), MBP (mouse; 1:1000; Covance; CN: 836504), MOG (mouse; 1:3000; Millipore; CN: MAB5680), PLP (rat; 1:500; Hybridoma AA3-PLP/DM20), vimentin (mouse; 1:1000; BD Pharmingen; CN: 550513), and α-tubulin (mouse; 1:10,000; Proteintech; CN: 660311).

### Immunohistochemistry

Forty days after EAE induction, subjects were injected with the anesthetic isoflurane and then perfused with 4% paraformaldehyde (PFA) solution in PBS via the left ventricle of the heart. Brains and spinal cords (encased in vertebral column) were postfixed overnight in 4% PFA at 4 °C. Lumbar spinal tissue was dissected from vertebrae, added to 30% sucrose solution (6 g sucrose, 18 mL Milli-Q purified water, 2 mL of 10 × PBS) for 2–3 days until fully permeated and then frozen at − 80 °C. Brains were sectioned at a thickness of 50 μm along the sagittal plane using a vibratome (Leica Biosystems, VT1000-S), while coronal spinal cord slices (30 μm each) were obtained using a cryostat set to − 20 °C. Both free-floating vibratome sections and cryosections were treated with antigen retrieval buffer (0.1 M citric acid, 10% ethanol) at 98 °C and then incubated in blocking buffer (2% normal goat serum, 1% Triton X-100 in PBS) for at least 2 h at room temperature followed by an overnight incubation with primary antibodies at 4 °C. The following day sections were washed in PBS and then incubated at room temperature for 2 h with Cy3-, Cy5-, or Alexa 488-conjugated secondary antibodies (1:400; Jackson ImmunoResearch; mouse Cy3 CN: 115–165–146; rabbit Cy3 CN: 111–165–144; mouse Cy5 CN: 115–175–146; rabbit Cy5 CN: 111–175–144; rat Cy5 CN: 112–175–167). The nuclear dye DAPI (Life Technologies) was then applied as a counterstain prior to a final rinse in PBS. Sections were then mounted onto microscope slides (Superfrost Plus, Fisher) with coverslips and mounting medium (Aquamount, ThermoFisher Scientific). The primary antibodies used for this technique were against the following antigens: CC1 (mouse; 1:300; Calbiochem; CN: OP80-100UG), GFAP (rabbit; 1:1000; Dako; CN: Z0334), MAP2 (mouse; 1:200; Millipore; CN: MAB3418), CD45 (rat; 1:200, Millipore; CN: CBL1326), Iba1 (rabbit; 1:500; Wako; CN: 019-19741), MBP (mouse; 1:400; Covance; CN: 836504), PLP (rat; 1:200; Hybridoma AA3–PLP/DM20), Olig2 (rabbit; 1:500; Millipore; CN: AB9610), and NeuN (mouse; 1:200, Millipore; CN: MAB377). Staining intensity was analyzed in the olfactory bulb, corpus callosum, hippocampus and cerebellum, while all white and gray matter portions of the lumbar spinal cord were assessed for staining intensity and positive cell density. MetaMorph software (molecular devices) was used to calculate integrated fluorescence intensity—the product of the area and mean pixel intensity. All quantifications involved pooling results from at least four brains, or spinal cords, per experimental condition and were completed blind to the samples’ genotype.

### Black Gold II myelin staining

The Black Gold II staining kit (Millipore; CN: AG105) was used in congruence with the manufacturer’s instructions outlined by Cheli et al. [4]. PFA-fixed lumbar spinal cord sections (30 μm each) were mounted onto Superfrost Plus slides (Fisher Scientific), air-dried, rehydrated in distilled water, and then transferred to a 0.3% Black Gold II solution (Millipore) for 10 min. Next, slides were washed twice for 2 min in distilled water and then submerged in a 1% sodium thiosulfate solution at 60 °C. Finally, slides were rinsed thrice in distilled water, dehydrated, immersed in xylene, and protected with a coverslip and Permount. Demyelinated lesions in the spinal cord were measured as a percentage of total white matter area using MetaMorph Software (Molecular Devices). Data represent results pooled from at least four spinal cords per experimental condition and ten slices per spinal cord.

### Statistical analysis

EAE data were analyzed by non-parametric tests. Group comparisons were performed with Mann–Whitney *U* test and the correlation coefficient (*r*^2^) was calculated by Pearson’s test. Using a confidence interval of 95%, the unpaired *t* test (Student’s *t* test) was used for all single between-group comparisons and multiple comparisons were investigated by one-way ANOVA followed by Bonferroni’s multiple comparison test to detect pairwise between-group differences. GraphPad Prism software was utilized for all statistical analyses. The criterion for reliable differences between groups was a fixed value of *p* < 0.05 for two-tailed tests and data are displayed as mean ± SEM. A minimum of four animals per experimental condition were compared for all morphological and biochemical endpoints, while at least 12 animals per condition were compared for behavioral assays.

## Results

### Conditional deletion of Cav1.2 channels in astrocytes during EAE

**Fig. 1:**
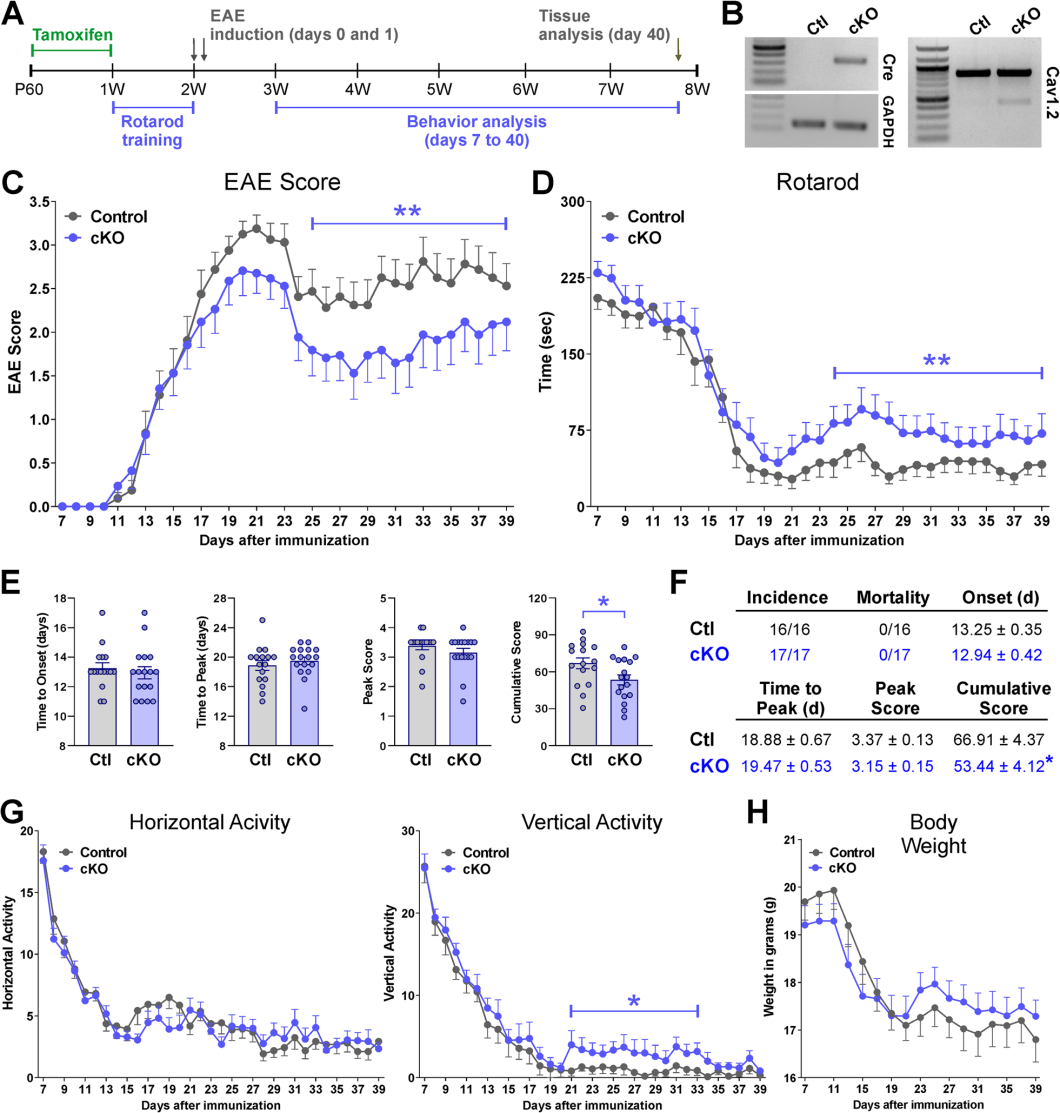
Decreased disease severity and motor deficits in Cav1.2^KO^ mice. **A** Tamoxifen was administered to Cav1.2^KO^ and control mice at P60. Subjects were trained on a rotarod before EAE induction. EAE was induced at D0, and daily behavioral testing was conducted from D7 to D40. **B** RT-PCR experiments for *Cre* and Cav1.2 performed with total RNA isolated from control and Cav1.2^KO^ lumbar spinal cords. **C** Disease severity of controls (gray) and Cav1.2^KO^ mice (blue) immunized for EAE was assessed daily. Clinical score was determined according to the criteria defined in the text. **D** Controls and Cav1.2^KO^ mice were evaluated using the rotarod performance test starting 1 week after EAE immunization. Speed of rotation gradually increased from 4 to 40 rpm in a 5 min interval. Latency to fall off the apparatus was measured daily and recorded in seconds. **E** Time to disease onset, time-to-peak disease, peak disease score, and cumulative disease score. Each dot denotes the mean of one subject. **F** Tabular presentation including disease incidence, mortality, average disease onset (EAE score ≥ 1), time-to-peak disease, peak disease score, and cumulative clinical EAE scores in control and Cav1.2^KO^ mice. **G** Horizontal and vertical motor activity of controls and Cav1.2^KO^ mice were evaluated daily and recorded as the frequency of passes through the midline of an open cage and how often a subject reared on their hind limbs, respectively. **H** Body weight was assessed every other day and recorded in grams. Group comparisons in **C**, **D**, **G** and **H** were performed with the Mann–Whitney *U* test. The unpaired *t* test was used for comparisons between experimental groups in **E** and **F**. At least 16 mice per condition were tested and values are presented as mean ± SEM *p < 0.05, **p < 0.01 versus control

We generated a conditional knock-out mouse for astrocytic Cav1.2 channels by cross breeding the *floxed* mutant Cav1.2 mouse [35] with the Glast1-*Cre*ER^T2^ transgenic line. Glast1-*Cre*ER^T2^ transgenic mice express a *Cre* recombinase activated by tamoxifen and controlled by the mouse Glast1 promoter, and genomic recombination occurs exclusively in astrocytes throughout most CNS regions [33]. We previously demonstrated a successful conditional deletion of Cav1.2 channels in astrocytes using several *Cre* lines both in vitro and in vivo [5, 38]. To induce *Cre* activity in Glast1-positive astrocytes, tamoxifen was injected intraperitoneally every day for 5 days starting 2 weeks before EAE induction (Fig. 1A). Both Cav1.2^KO^ mice (Cav1.2^f/f^, Glast1*Cre*^±^) and *floxed* controls (Cav1.2^f/f^, Glast1*Cre*^−/−^) were injected with tamoxifen and, importantly, neither body mass nor rotarod performance were significantly different between genotypes prior to EAE immunization (data not shown). Recombination was evaluated in RNA samples isolated from control and Cav1.2^KO^ lumbar spinal cords (Fig. 1B). The presence of the mRNA for the *Cre* enzyme and the truncated version of the Cav1.2 transcript demonstrated a successful genomic recombination in Cav1.2^KO^ subjects (Fig. 1B).

**Fig. 2:**
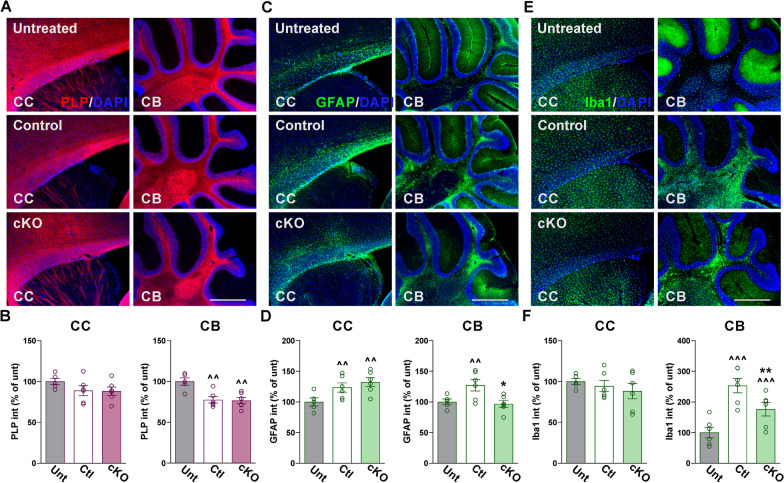
Decreased neuroinflammation in the Cav1.2^KO^ cerebellum. **A**, **C**, **E** Representative sagittal sections of brains immunostained for PLP, GFAP, and Iba1. Tissue was collected from untreated, control, and Cav1.2^KO^ mice at D39. Scale bar, 180 μm. **B**, **D**, **F** PLP, GFAP, and Iba1 expression was quantified by analyzing the integrated fluorescence intensity in the corpus callosum (CC) and cerebellum (CB). One-way ANOVA followed by the Bonferroni’s test was used for comparisons between experimental groups. Analysis consisted of at least four brains per condition and each dot denotes the mean of one subject. Values are presented as mean ± SEM ^^*p* < 0.01, ^^^*p* < 0.001 versus untreated; *p < 0.05, **p < 0.01 versus control

No significant differences in EAE score and motor activity were observed between experimental conditions during either the onset of symptoms or peak of the disease (Fig. 1C–G). Controls and Cav1.2^KO^ mice exhibited similar decreases in activity and body weight throughout the EAE acute phase (Fig. 1G, H). Both experimental groups also reached peak clinical score in the same number of day post-immunization and exhibited similar maximum scores (Fig. 1C, E, F). However, during the EAE chronic phase, we found reduced clinical scores in Cav1.2^KO^ mice compared to controls (Fig. 1C). This corresponded with a greater latency to fall off the rotarod, lower cumulative score, and improved vertical activity in Cav1.2^KO^ mice during that same period (Fig. 1D–G). Immunohistochemical experiments were performed to assess demyelination and neuroinflammation in the brain of EAE mice. Myelin proteins were first analyzed in several brain regions, such as the olfactory bulb, corpus callosum, hippocampus, and cerebellum. Controls and Cav1.2^KO^ animals showed normal levels of proteolipid protein (PLP) expression in all brain areas except for the cerebellum, where there was a significant reduction in PLP for both groups 40 days after EAE induction (Fig. 2A, B, all data not shown). Furthermore, controls and Cav1.2^KO^ animals showed significant increases in the expression of glial fibrillary acidic protein (GFAP) in the corpus callosum, and cerebellum compared to untreated subjects (Fig. 2C, D). Microglial reactivity—analyzed using the antibody ionized calcium-binding adapter molecule 1 (Iba1)—was also increased in the cerebellar white matter of controls and Cav1.2^KO^ mice (Fig. 2E, F). These findings suggest that EAE enhances neuroinflammation throughout the brain, which in turn causes substantial cerebellar demyelination. Although Cav1.2^KO^ mice showed a similar reduction in PLP expression in the cerebellum as controls (Fig. 2A, B), the fluorescence intensity of both GFAP and Iba1 was significantly reduced in these animals (Fig. 2C–F).

**Fig. 3:**
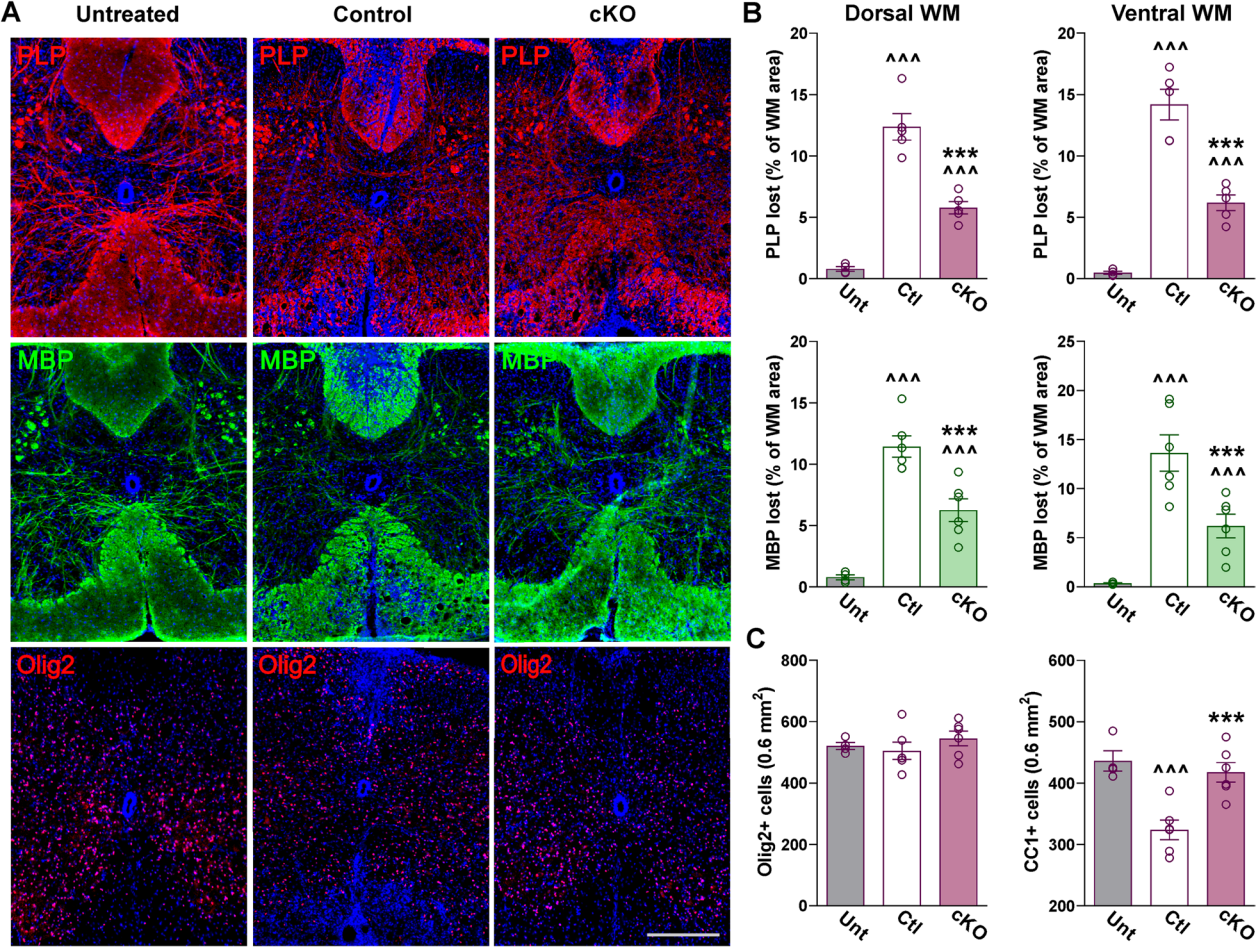
Reduced demyelination in the Cav1.2^KO^ spinal cord. **A** Representative coronal sections of lumbar spinal cords immunostained for PLP, MBP, and Olig2. Tissue was collected from untreated, control, and Cav1.2^KO^ mice at D39. Scale bar, 90 μm. **B** Demyelinated lesions were measured as a percentage of the dorsal and ventral white matter (WM) area using PLP and MBP. **C **Olig2- and CC1-positive cell density were measured in the entire spinal section. One-way ANOVA followed by the Bonferroni’s test was used for comparisons between experimental groups. Analysis consisted of at least four spinal cords per condition and each dot denotes the mean of one subject. Values are presented as mean ± SEM ^^^*p* < 0.001 versus untreated; ***p < 0.001 versus control

**Fig. 4:**
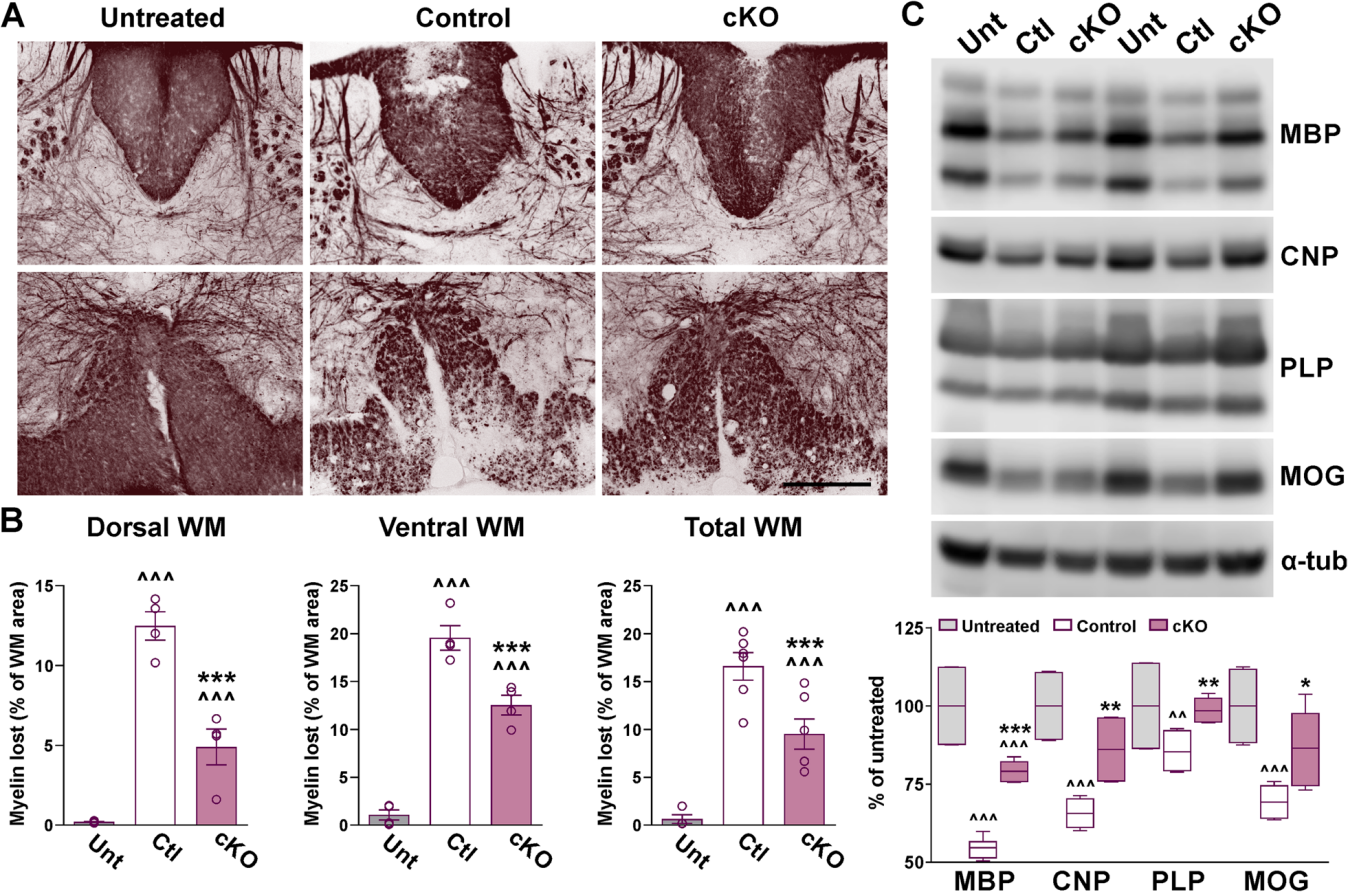
Myelin survival in the Cav1.2^KO^ spinal cord. **A** Black Gold II staining in representative coronal sections of lumbar spinal cords. Tissue was collected from untreated, control and Cav1.2^KO^ mice at D39. Scale bar, 90 μm. **B** Demyelinated lesions were measured as a percentage of the dorsal, ventral, and total WM area. **C** Representative western blots for MBP, CNP, PLP, and MOG in the lumbar spinal cord of untreated, control, and Cav1.2^KO^ mice. α-Tubulin was used as internal standard. One-way ANOVA followed by the Bonferroni’s test was used for comparisons between experimental groups. Analysis consisted of at least four spinal cords per condition and each dot denotes the mean of one subject. Values are presented as mean ± SEM ^^*p* < 0.01, ^^^*p* < 0.001 versus untreated; *p < 0.05, **p < 0.01, ***p < 0.001 versus control

Demyelination and neuroinflammation were evaluated by immunohistochemistry within the gray and white matter areas of the lumbar spinal cord 40 days after EAE induction. The amount of myelin lost was calculated as the percentage of the total white matter area that was devoid of PLP and MBP staining. In control subjects, ~ 12–14% of the dorsal and ventral white matter regions showed an absence of PLP and MBP immunostaining (Fig. 3A, B). Importantly, the degree of demyelination was lessened in Cav1.2^KO^ spinal cords (Fig. 3A, B). Only ~ 5% of the dorsal and ventral white matter areas showed a lack of MBP and PLP signal in Cav1.2^KO^ mice (Fig. 3A, B). No differences were found between controls, Cav1.2^KO^, and untreated mice in the total number of oligodendrocytes, as determined by number of oligodendrocyte transcription factor (Olig2)-positive cells (Fig. 3A, C). However, compared to untreated mice the density of mature myelinating CC1-positive oligodendrocytes was significantly reduced in controls and Cav1.2^KO^ samples (Fig. 3C). In line with the aforementioned results, the density of CC1-positive cells in Cav1.2^KO^ spinal cords was higher than that of controls and similar to untreated levels (Fig. 3C). These data suggest an enhanced survival of mature-myelinating oligodendrocytes in Cav1.2^KO^ spinal cords. Changes in myelination were verified by Black Gold myelin staining (Fig. 4A, B) and western blotting for the myelin proteins MBP, CNP, PLP, and MOG (Fig. 4C). Relative to controls, demyelinated areas identified by the absence of Black Gold staining were reduced ~ 50% in Cav1.2^KO^ samples (Fig. 4A, B). Western blot analysis performed with total proteins isolated from the lumbar section of the spinal cord also revealed a significant attenuation of myelin degeneration in Cav1.2^KO^ subjects (Fig. 4C). Relative to untreated animals the expression of MBP, CNP, PLP, and MOG in control spinal cords was reduced by 40% on average; in contrast, Cav1.2^KO^ samples displayed only a ~ 20% decline across myelin proteins (Fig. 4C).

**Fig. 5:**
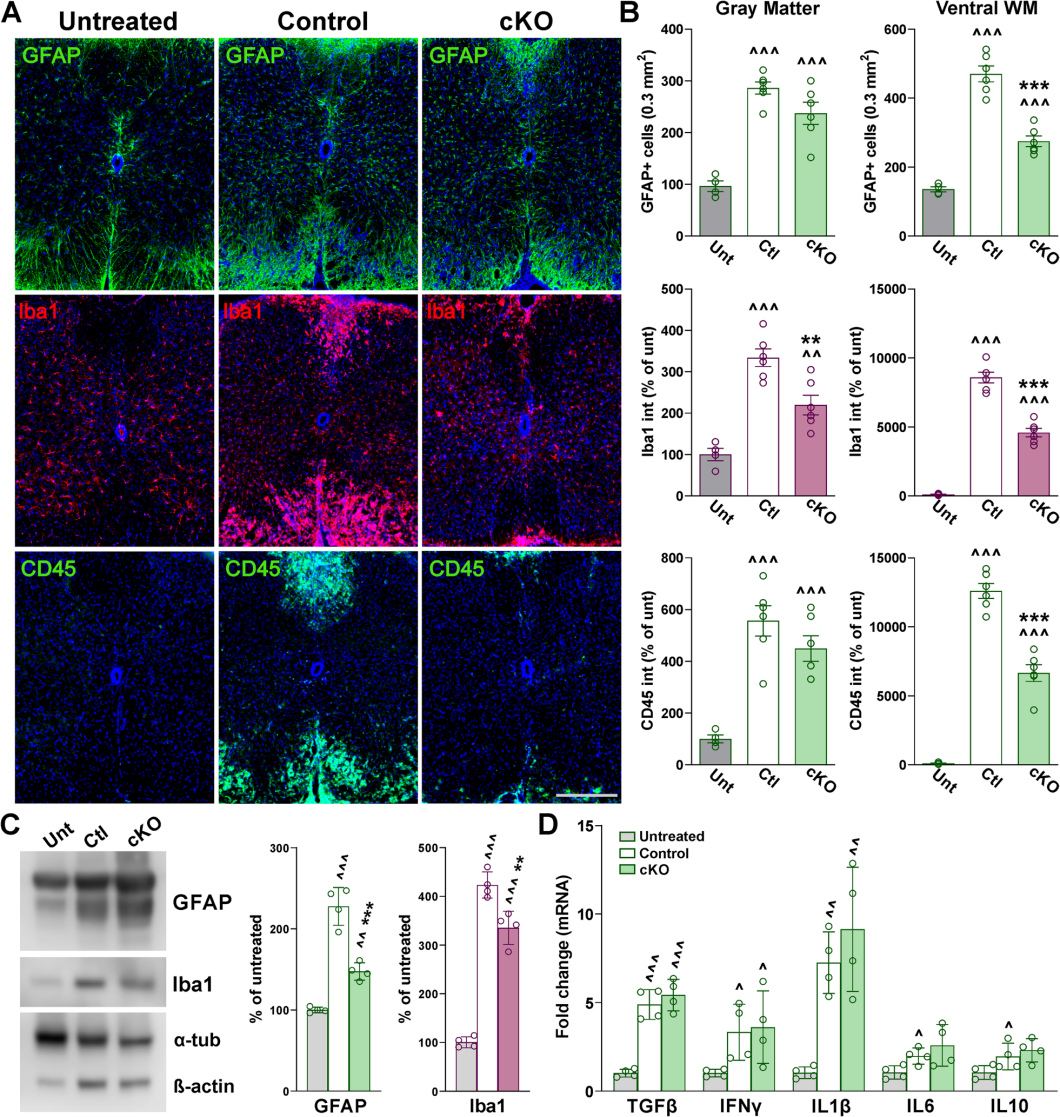
Reduced astrocyte and microglia reactivity and lymphocyte infiltration in the Cav1.2^KO^ spinal cord. **A** Representative coronal sections of lumbar spinal cords immunostained for GFAP, Iba1, and CD45. Tissue was collected from untreated, control, and Cav1.2^KO^ mice at D39. Scale bar, 90 μm. **B** Density of GFAP-positive cells and integrated fluorescence intensity of Iba1 and CD45 were quantified in the spinal gray matter and ventral white matter (WM). **C** Representative western blots for GFAP and Iba1 in the lumbar spinal cord of untreated, control, and Cav1.2^KO^ mice. α-Tubulin and β-actin were used as internal standards. **D** qPCR analysis of TGFβ, IFNγ, IL1β, IL6, and IL10 in spinal cord samples from untreated, control, and Cav1.2^KO^ mice at D39. GAPDH and TBP were used as internal standards and values are expressed as fold change of untreated values ± SEM. One-way ANOVA followed by the Bonferroni’s test was used for comparisons between experimental groups. Analysis consisted of at least four spinal cords per condition and each dot denotes the mean of one subject. Values are presented as mean ± SEM ^*p* < 0.05, ^^*p* < 0.01, ^^^*p* < 0.001 versus untreated; **p < 0.01, ***p < 0.001 versus control

**Fig. 6:**
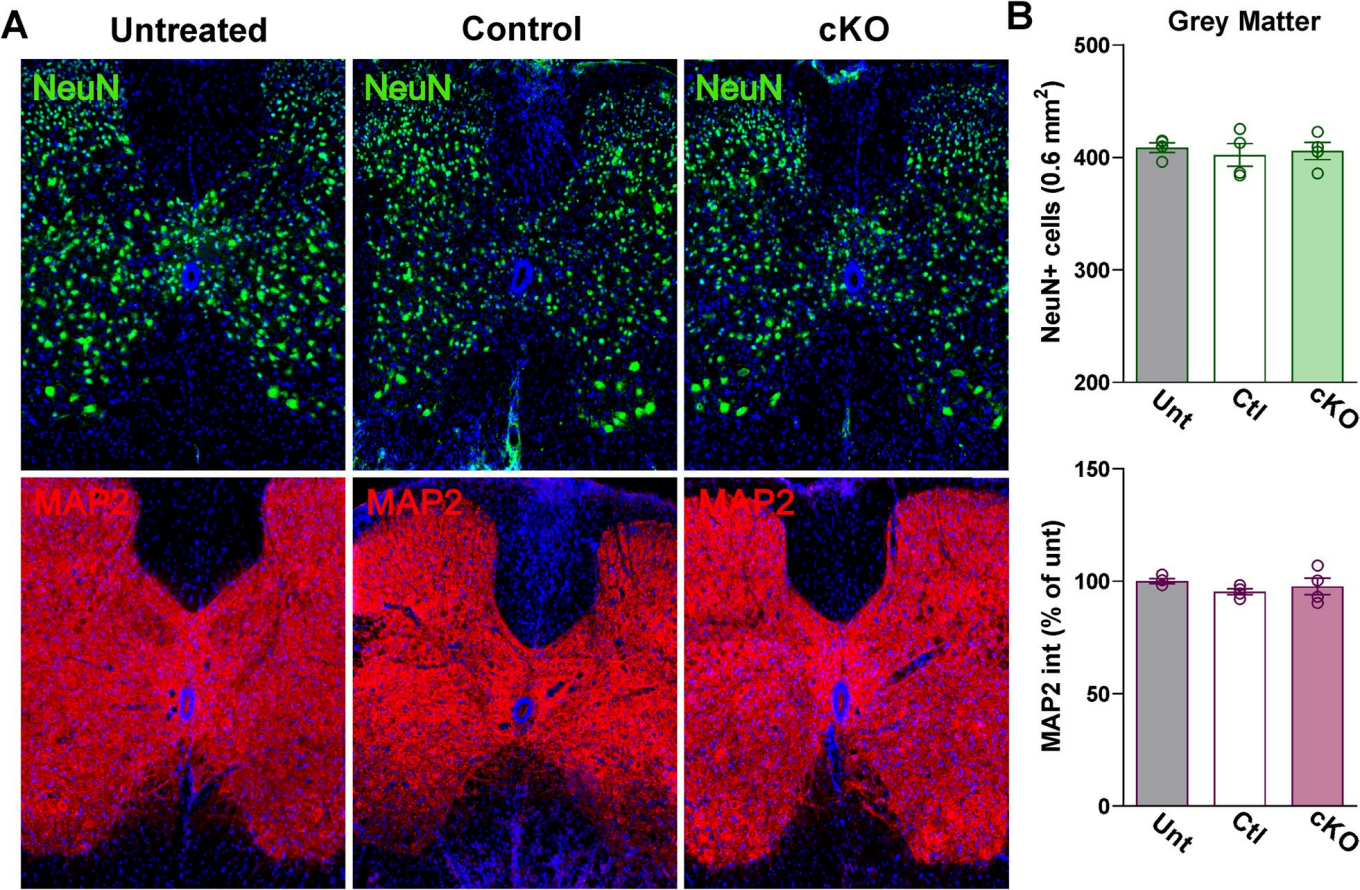
Absence of neuronal changes in the Cav1.2^KO^ spinal cord. **A** Representative coronal sections of lumbar spinal cords immunostained for NeuN and MAP2. Tissue was collected from untreated, control, and Cav1.2^KO^ mice at D39. Scale bar, 90 μm. **B** Density of NeuN-positive cells and integrated fluorescence intensity of MAP2 were quantified in the spinal gray matter. One-way ANOVA followed by the Bonferroni’s test was used for comparisons between experimental groups. Analysis consisted of at least four spinal cords per condition and each dot denotes the mean of one subject. Values are presented as mean ± SEM

Neuroinflammation in EAE spinal cords was demonstrated by robust increases in the density of GFAP-positive cells and expression of Iba1 and CD45—an antigen highly expressed in lymphocytes recruited to lesion sites following astrogliosis (Fig. 5A, B). This was corroborated by western blotting for GFAP and Iba1 (Fig. 5C). In addition, the expression of several pro-inflammatory cytokines was evaluated by qPCR in the lumbar spinal cord (Fig. 5D). These experiments revealed that the mRNAs for the pro-inflammatory factors TGFβ, IFNγ, IL1β, IL6, and IL10 were greatly increased in controls and Cav1.2^KO^ samples (Fig. 5D). Despite no changes in the levels of pro-inflammatory cytokines between controls and Cav1.2^KO^ mice (Fig. 5D), we did find fewer GFAP-positive cells and reduced expression of Iba1 and CD45 in Cav1.2^KO^ animals (Fig. 5A, B). This attenuation of neuroinflammation-related cells was primarily concentrated in the central region of the ventral white mater—the spinal cord area most affected by demyelination (Fig. 5A, B). Western blot analysis for GFAP and Iba1 also revealed a significant attenuation of astrocytes and microglia activation in Cav1.2^KO^ spinal cords relative to controls (Fig. 5C). Immunohistochemical experiments were also completed to evaluate neurodegeneration in the spinal cord of controls and Cav1.2^KO^ animals. NeuN and MAP2 were used to measure neuronal numbers and morphology, respectively. No signs of neurodegeneration and/or neuronal loss were found in the gray matter of the lumbar spinal cord at the end of the EAE protocol; the number of NeuN-positive cells, as well as MAP2 levels, were normal across experimental groups (Fig. 6A, B). Therefore, ablating astrocytic Cav1.2 channels prior to EAE induction has a considerable influence on astrocyte and microglia activation, peripheral immune cell infiltration, and myelin survival in the spinal cord.

### Pharmacological antagonism of Cav1.2 channels during EAE

**Fig. 7:**
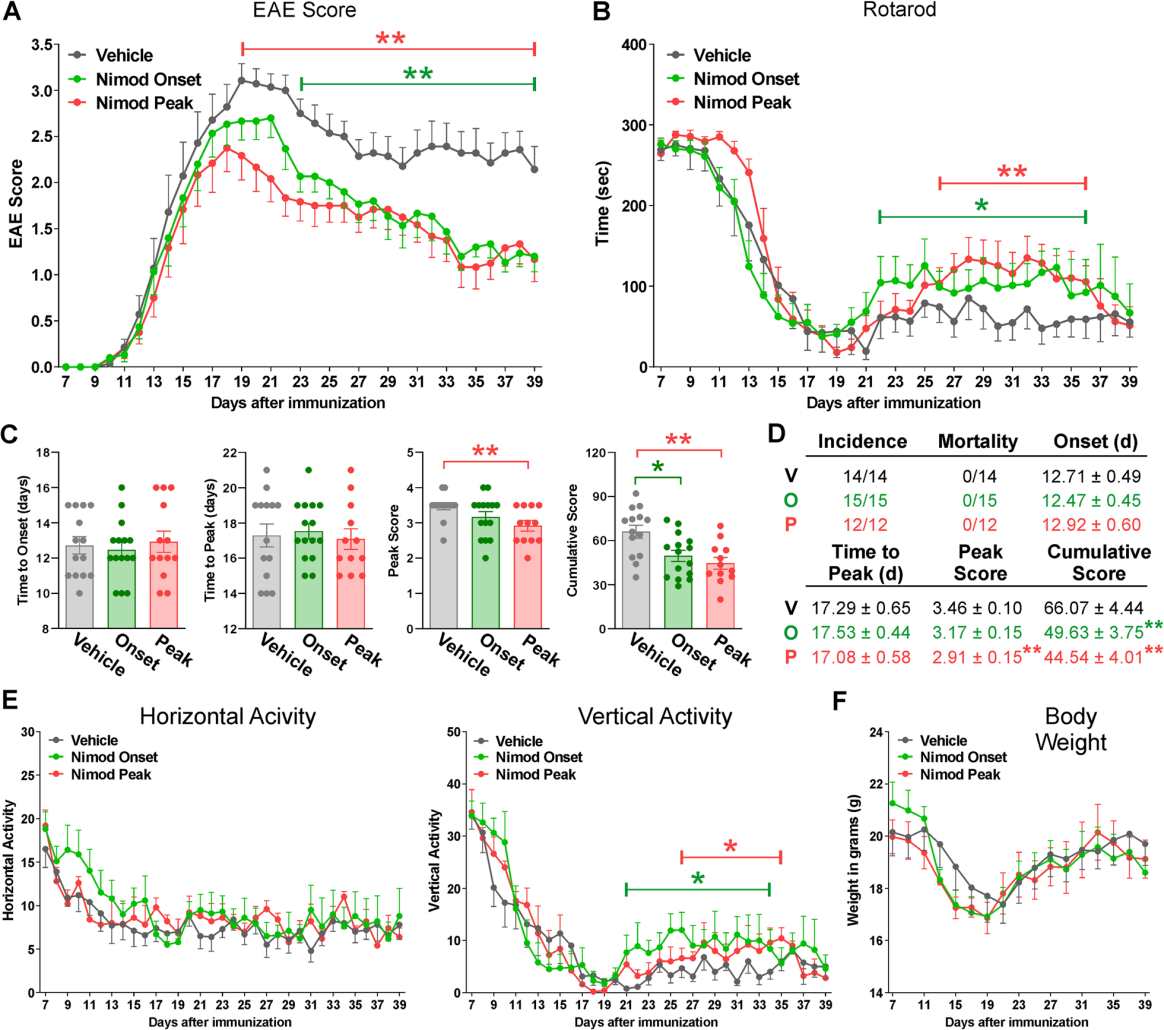
Decreased disease severity and motor deficits in nimodipine-treated EAE mice. **A** Disease severity of EAE mice treated with vehicle (gray) and nimodipine during the acute (D7–17) (green) or peak (D14–24) (red) phase of the disease was assessed daily. Clinical score was determined according to the criteria defined in the text. **B** Vehicle- and nimodipine-treated mice were evaluated using the rotarod performance test starting 1 week after EAE immunization. Speed of rotation gradually increased from 4 to 40 rpm in a 5 min interval. Latency to fall off the apparatus was measured daily and recorded in seconds. **C** Time to disease onset, time-to-peak disease, peak disease score, and cumulative disease score. Each dot denotes the mean of one subject. **D** Tabular presentation including disease incidence, mortality, average disease onset (EAE score ≥ 1), time-to-peak disease, peak disease score, and cumulative clinical EAE scores in vehicle- and nimodipine-treated mice. **E** Horizontal and vertical motor activity of vehicle- and nimodipine-treated mice were evaluated daily and recorded as the frequency of passes through the midline of an open cage and how often a subject reared on their hind limbs, respectively. **F** Body weight was assessed every other day and recorded in grams. Group comparisons in **A**, **B**, **E** and **F** were performed with the Mann–Whitney *U* test. One-way ANOVA followed by the Bonferroni’s test was used for comparisons between experimental groups in **C** and **D**. At least 12 mice per condition were tested and values are presented as mean ± SEM *p < 0.05, **p < 0.01 versus vehicle

We blocked CNS Cav1.2 channels using nimodipine, a VGCC antagonist with high affinity for the brain and spinal cord [18]. Vehicle or nimodipine was administered subcutaneously every other day either during the onset (D7–17) or peak phase (D14–24) of EAE. Behavioral phenotypes remained consistent across experimental groups during the EAE acute phase (Fig. 7A, B, E, F). Time to the onset of symptoms and peak disease were similar between vehicle and nimodipine-treated subjects (Fig. 7A, C, D). However, compared to vehicle-treated mice, animals that received nimodipine during the onset or peak phases of EAE exhibited reduced disease severity at the finale of the peak and chronic phases (Fig. 7A), as well as a transient improvement in rotarod performance and vertical activity during the chronic phase (Fig. 7B, E). In addition, nimodipine administration at the peak significantly reduced maximum and cumulative scores, whereas nimodipine treatment throughout the onset of symptoms decreased the summation of scores (Fig. 7C, D). Importantly, no statistical differences were detected within vehicle administration protocols in any behavioral determination. Thus, results from vehicle injected animals during the onset and peak were combined into a single experimental group to simplify the presentation of the data. In agreement with our knockout experiments, these findings suggest that Cav1.2 channels moderate some motor deficits during the chronic phase of EAE.

**Fig. 8:**
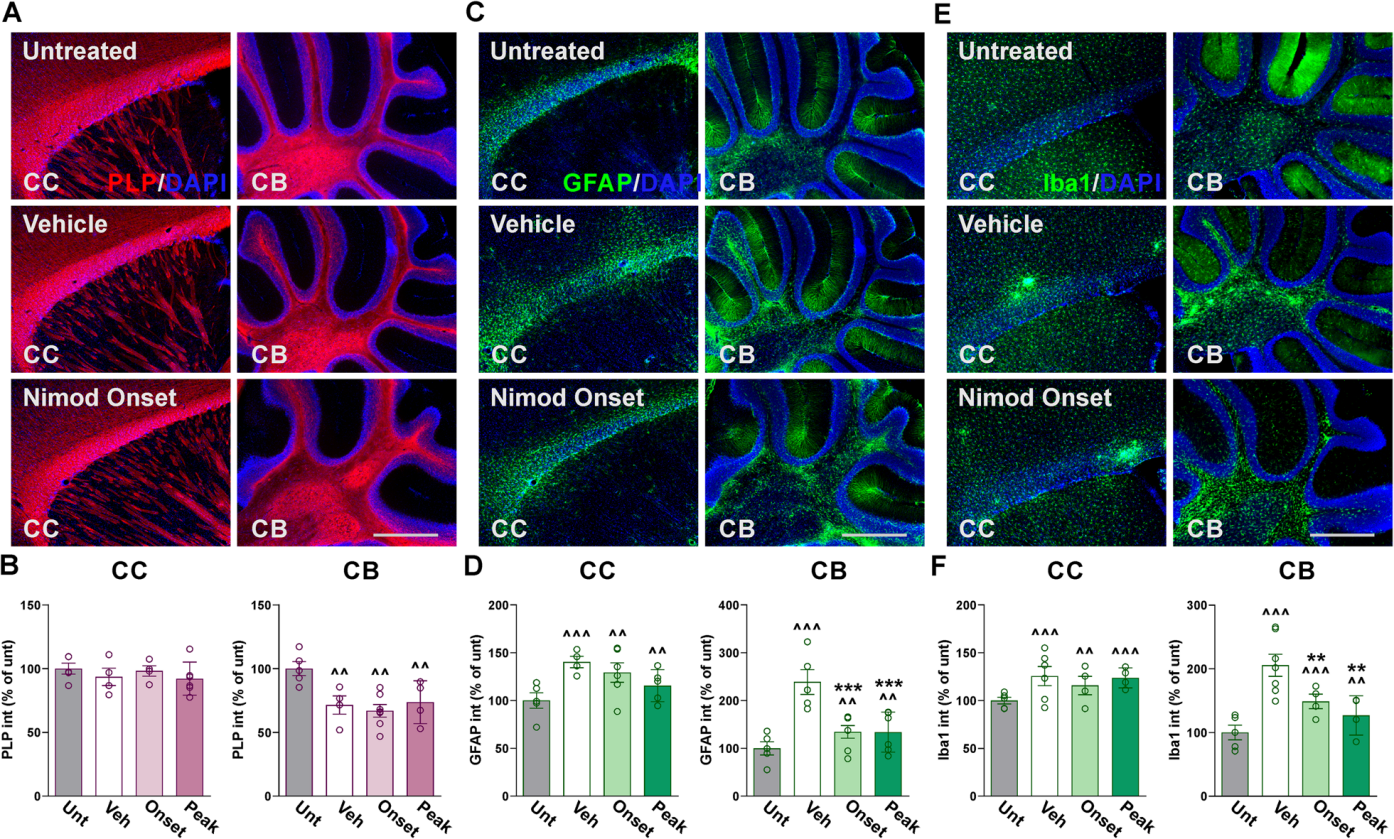
Decreased neuroinflammation in the nimodipine-treated cerebellum. **A**, **C**, **E** Representative sagittal sections of brains immunostained for PLP, GFAP, and Iba1. Tissue was collected from untreated subjects and EAE mice treated with vehicle or nimodipine during the acute or peak phase of the disease. Scale bar, 180 μm. **B**, **D**, **F** PLP, GFAP, and Iba1 expression was quantified by analyzing the integrated fluorescence intensity in the corpus callosum (CC) and cerebellum (CB). One-way ANOVA followed by the Bonferroni’s test was used for comparisons between experimental groups. Analysis consisted of at least four brains per condition and each dot denotes the mean of one subject. Values are presented as mean ± SEM ^^*p* < 0.01, ^^^*p* < 0.001 versus untreated; **p < 0.01, ***p < 0.001 versus vehicle

The effects of phase-dependent nimodipine administration on the CNS of EAE mice were assessed via immunohistochemistry in select brain areas and spinal cord sections. Compared to untreated subjects, PLP expression decreased substantially in the cerebellum of vehicle- and nimodipine-treated animals (Fig. 8A, B), while the fluorescence intensity of GFAP and Iba1 was exacerbated in the corpus callosum and cerebellum of these experimental groups (Fig. 8C–F). When nimodipine was administered during the onset and peak phases of EAE, GFAP and Iba1 expression remained consistent with vehicle levels in the corpus callosum (Fig. 8A, B), even though both nimodipine treatments were equally effective in reducing the expression of GFAP and Iba1 in cerebellar white mater (Fig. 8C–F). In concordance with our findings in the Cav1.2^KO^ brain, pharmacologically inhibiting Cav1.2 channels, irrespective of administration time, effectively ameliorates astrocyte and microglial reactivity in the cerebellum.

**Fig. 9:**
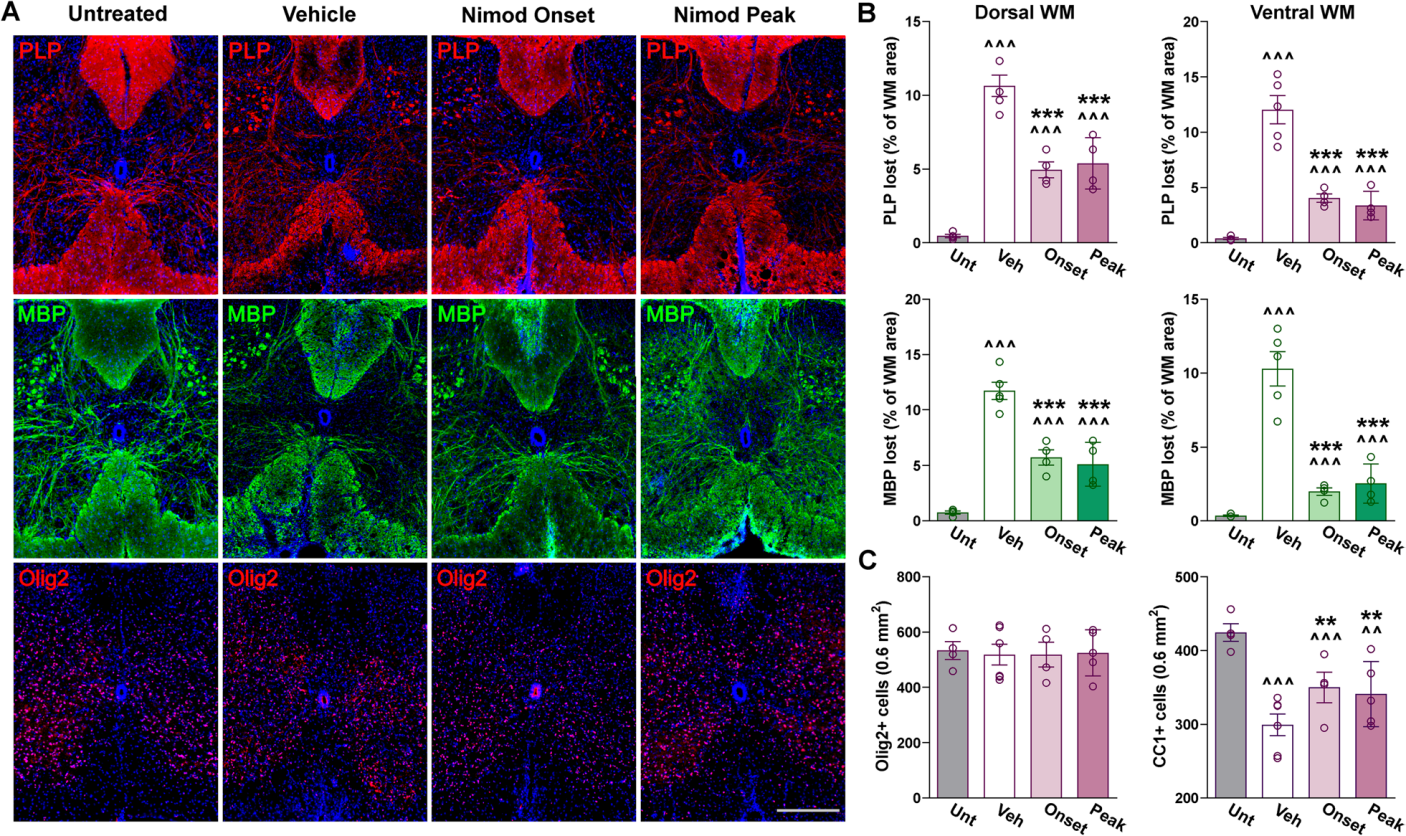
Reduced demyelination in the nimodipine-treated spinal cord. **A** Representative coronal sections of lumbar spinal cords immunostained for PLP, MBP, and Olig2. Tissue was collected from untreated subjects and EAE mice treated with vehicle or nimodipine during the acute or peak phase of the disease. Scale bar, 90 μm. **B** Demyelinated lesions were measured as a percentage of the dorsal and ventral WM area using PLP and MBP. **C** Olig2- and CC1-positive cell density were measured in the entire spinal section. One-way ANOVA followed by the Bonferroni’s test was used for comparisons between experimental groups. Analysis consisted of at least four spinal cords per condition and each dot denotes the mean of one subject. Values are presented as mean ± SEM ^^*p* < 0.01, ^^^*p* < 0.001 versus untreated; **p < 0.01, ***p < 0.001 versus vehicle

**Fig. 10:**
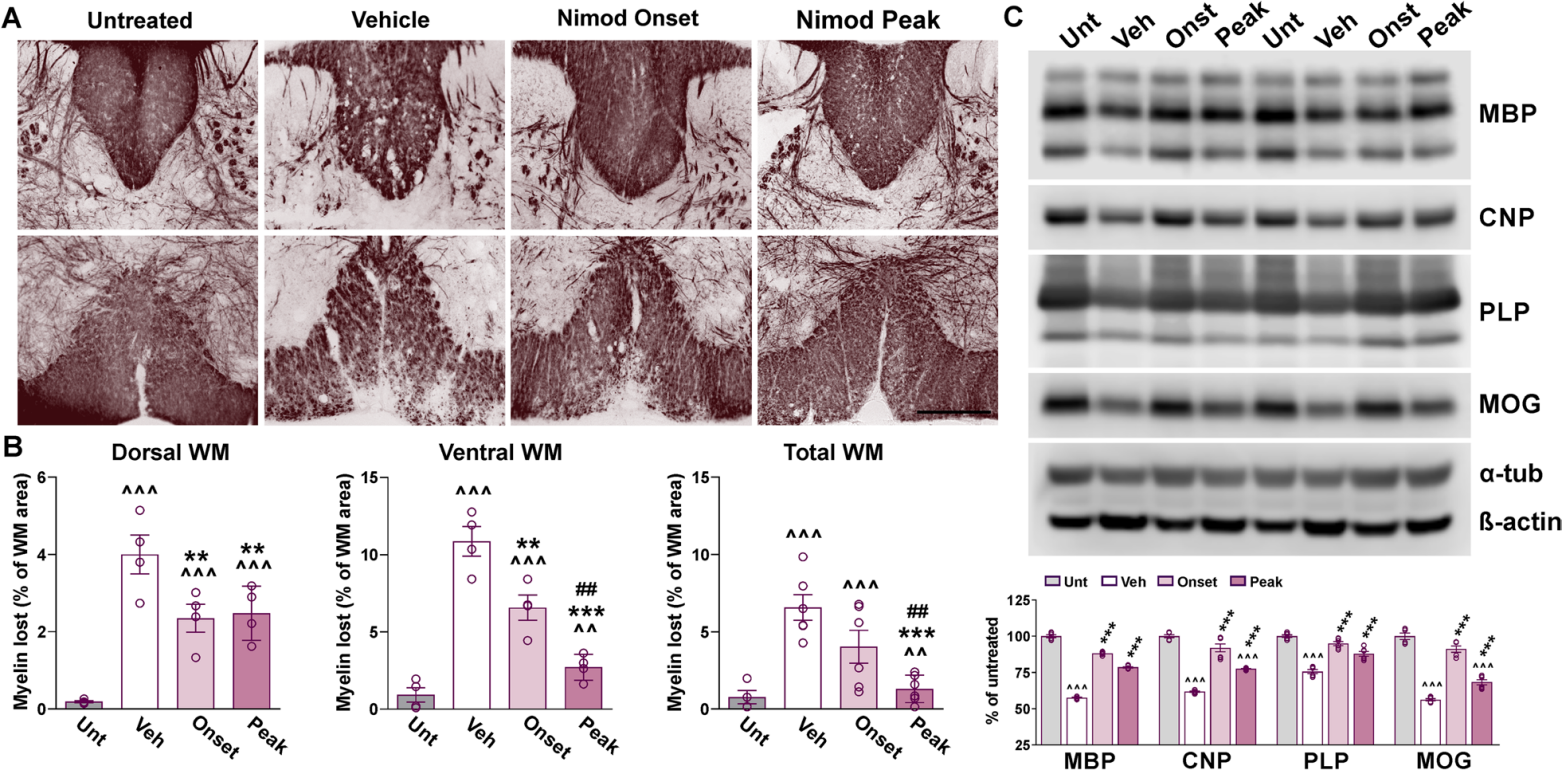
Myelin survival in the nimodipine-treated spinal cord. **A** Black Gold II staining in representative coronal sections of lumbar spinal cords. Tissue was collected from untreated subjects and EAE mice treated with vehicle or nimodipine during the acute or peak phase of the disease. Scale bar, 90 μm. **B** Demyelinated lesions were measured as a percentage of the dorsal, ventral, and total WM area. **C** Representative western blots for MBP, CNP, PLP, and MOG in the lumbar spinal cord of untreated subjects and EAE mice treated with vehicle or nimodipine during the acute or peak phase of the disease. α-Tubulin and β-actin were used as internal standards. One-way ANOVA followed by the Bonferroni’s test was used for comparisons between experimental groups. Analysis consisted of four spinal cords per condition and each dot denotes the mean of one subject. Values are presented as mean ± SEM ^^*p* < 0.01, ^^^*p* < 0.001 versus untreated; **p < 0.01, ***p < 0.001 versus vehicle; ##*p* < 0.01 versus onset

Relative to untreated animals, we found a significant loss of PLP and MBP expression in white matter areas of vehicle- and nimodipine-treated spinal cords (Fig. 9A, B). Importantly, both PLP and MBP losses were diminished by nimodipine administration during the onset and peak phases of EAE (Fig. 9A, B). These changes were particularly evident in the ventral white matter area of nimodipine-treated spinal cords, which showed less than a 5% loss of PLP and MBP (Fig. 9A, B). Correspondingly, the number of mature myelinating oligodendrocytes (CC1-positive cells) was promoted by both nimodipine treatments (Fig. 9C). Notably, both nimodipine schedules were equally efficient in preventing PLP, MBP and CC1-positive cells loss 40 days after EAE induction (Fig. 9A–C). Black Gold staining and western blotting also revealed significant decreases in spinal demyelination between vehicle- and nimodipine-treated mice (Fig. 10A–C). Based on these data, nimodipine administration during the EAE peak was the most efficient protocol in protecting total and ventral myelin in the spinal cord (Fig. 10A, B).

**Fig. 11:**
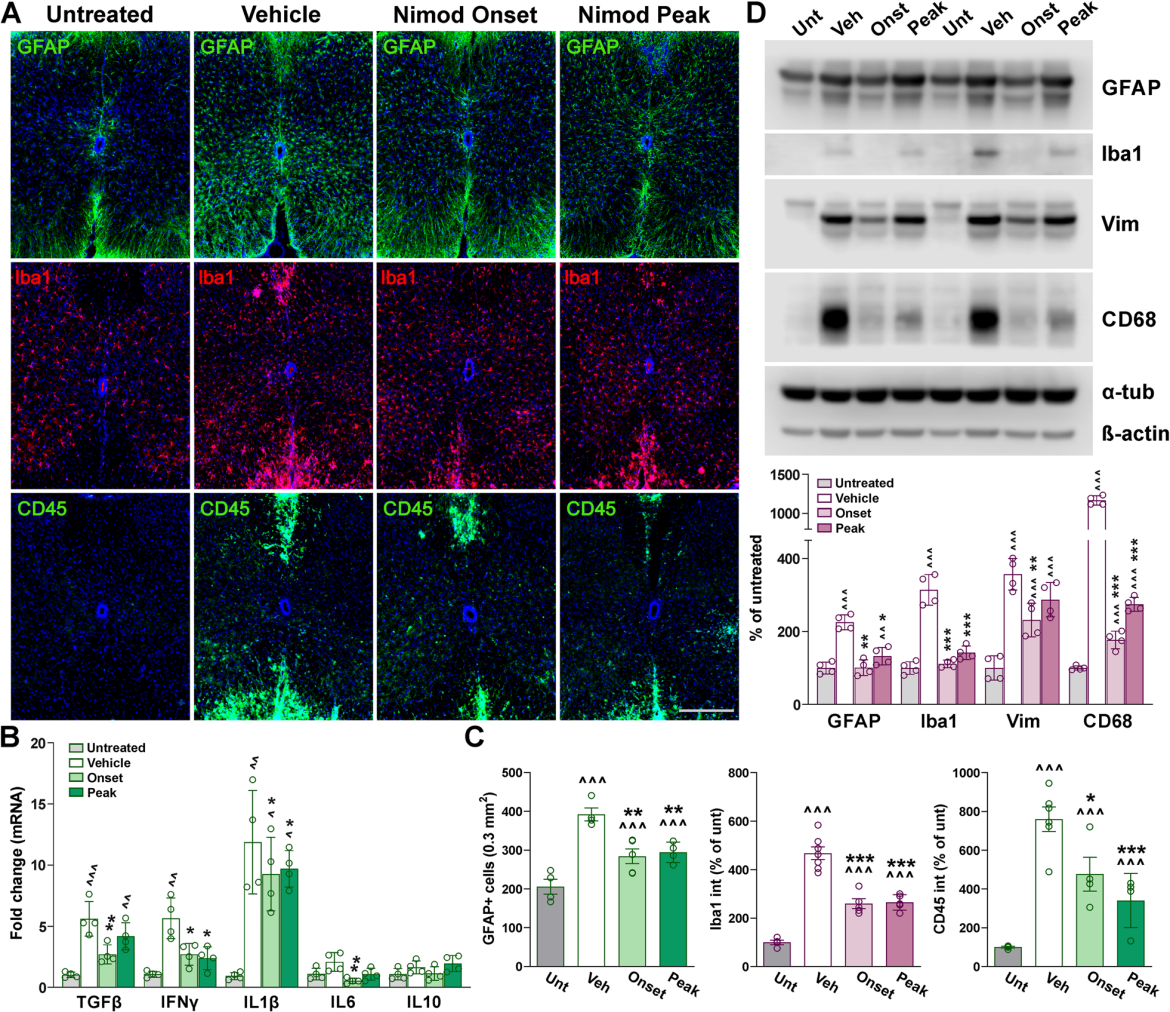
Reduced astrocyte and microglia reactivity and lymphocyte infiltration in the nimodipine-treated spinal cord. **A** Representative coronal sections of lumbar spinal cords immunostained for GFAP, Iba1, and CD45. Tissue was collected from untreated subjects and EAE mice treated with vehicle or nimodipine during the acute and peak phase of the disease. Scale bar, 90 μm. **B** qPCR analysis of TGFβ, IFNγ, IL1β, IL6, and IL10 in spinal cord samples from untreated subjects and EAE mice treated with vehicle or nimodipine during the acute and peak phase of the disease. GAPDH and TBP were used as internal standards and values are expressed as fold change of untreated values ± SEM. **C** Density of GFAP-positive cells and integrated fluorescence intensity of Iba1 and CD45 were quantified in the entire spinal section. **D** Representative western blots for GFAP, Iba1, vimentin, and CD68 in the lumbar spinal cord of untreated subjects and EAE mice treated with vehicle or nimodipine during the acute and peak phase of the disease. α-Tubulin and β-actin were used as internal standards. One-way ANOVA followed by the Bonferroni’s test was used for comparisons between experimental groups. Analysis consisted of at least four spinal cords per condition and each dot denotes the mean of one subject. Values are presented as mean ± SEM ^*p* < 0.05, ^^*p* < 0.01, ^^^*p* < 0.001 versus untreated; *p < 0.05, **p < 0.01, ***p < 0.001 versus vehicle

Furthermore, neuroinflammation was analyzed by determining the density of GFAP-positive cells and the expression of Iba1 and CD45 in both the white and grey matter areas of the lumbar spinal cord (Fig. 11A, C). The number of reactive astrocytes, as well as the fluorescent intensity for Iba1 and CD45, was substantially attenuated in the nimodipine-treated spinal cord relative to vehicle-injected animals (Fig. 11A, C). Similar to MBP and PLP, reductions in Iba1 and CD45 levels were prominent in the ventral white matter area (Fig. 11A). The level of the astrogliosis markers GFAP and vimentin (Vim) and the expression of the reactive microglia proteins Iba1 and CD68 were also found to be reduced in western blot experiments completed with spinal cord samples of nimodipine-treated animals (Fig. 11D). Importantly, spinal cord inflammation was reduced to a similar degree by both protocols of nimodipine administration (Fig. 11A, C, D). In addition, mRNAs for the pro-inflammatory factors TGFβ, IFNγ, IL1β, and IL6 were significantly decreased, particularly in mice treated with nimodipine during EAE onset (Fig. 11B). Finally, the density of NeuN-positive cells and fluorescent intensity for MAP2 in the gray matter region of vehicle- and nimodipine-treated spinal cords was equal to untreated levels (data not shown). Thus, consistent with our knockout findings, antagonizing Cav1.2 channels pharmacologically, either during EAE onset or peak, reduces myelin damage and neuroinflammation in the spinal cord.

## Discussion

### Ablating astrocytic Cav1.2 channels mitigates neuroinflammation and promotes myelin survival in EAE

Both in vitro and in vivo data confirm that astrocytes from several CNS regions express functional VGCCs. Cheli et al. [5] reported that cultured cortical astrocytes primarily express L-type channels and that Ca^++^ influx via L-type channels elicited an increase in intracellular Ca^++^ subsequent to membrane depolarization. In vivo studies have revealed that subventricular and thalamic astrocytes mediate Ca^++^ currents via membranous VGCCs [24, 37] and astrocytes of the ventrobasal thalamus exhibit VG Ca^++^ oscillations [17]. Corroboratively, Zamora et al. [38] found a significant rise in intracellular Ca^++^ in cortical astrocytes following membrane depolarization, which was abolished in Cav1.2 knockout mice. VGCCs are also upregulated in astrocytes under pathological conditions. For example, increased expression of L- and P/Q-type channels was found in hippocampal reactive astrocytes following the induction of status epilepticus [36]. L-type channel expression was also increased in reactive astrocytes of mice submitted to various brain injuries, such as mechanical lesions and ischemia [34]. Accordingly, Cheli et al. [5] showed significant L-type VGCC upregulation in reactive astrocytes following administration of LPS in cultured cortical astrocytes.

While astrocytic Cav1.2 channels have been identified heretofore as a salient modulator of astrogliosis, neuroinflammation, and demyelination in models of toxin-induced inflammation and myelin degradation [5, 38], we elucidated their role in the context of autoimmunity. Our results suggest that astrocytic Cav1.2 channels participate in the moderation of EAE severity. Astrocytic Cav1.2^KO^ mice exhibited improved disease symptomology during the EAE chronic phase, which coincided with better motor performance on the rotarod test and greater vertical activity. Histological analyses of the brain and spinal cord support these data; we found reduced astrocyte and microglia reactivity in the Cav1.2^KO^ cerebellum, the brain region most consistently targeted by MS and EAE [21]. In the Cav1.2^KO^ spinal cord, we demonstrated enhanced myelin survival through the protection of mature CC1-positive cells by reduced astrogliosis, lymphocyte infiltration, and microglia reactivity. These data indicate that Cav1.2^KO^ astrocytes are less functional, recruiting fewer reactive microglia and immune cells and, as a result, protect myelin.

Although astrocytes are typically seen as not electrically excitable, highly localized membrane depolarizations were recently detected in astrocyte microdomains, such as fine processes [1]. VGCCs such as Cav1.2 would be most relevant in these regions, and highly important in generating the cellular responses to depolarizing signals. Accordingly, the expression of VG channels is highly heterogenous among the microdomains of astrocytes [19, 20]. Therefore, it is possible that during neuroinflammation, Cav1.2 governs astrocyte reactivity through highly localized responses, such as communication with damaged oligodendrocytes and reactive microglia. Furthermore, Cav1.2 channels may promote astrogliosis through various biochemical alterations in quiescent astrocytes. Several processes that contribute to morphological changes in reactive cells, such as increased expression and phosphorylation of GFAP, are precipitated by Ca^++^ influx through Cav1.2 channels [12]. L-type channels can also influence gene transcription through nuclear Ca^++^-signaling pathways [2] and activate transcription factors and nuclear Ca^++^-dependent enzymes, such as CaMKIV [9, 11].

Interestingly, despite ablating Cav1.2 channels in astrocytes prior to EAE immunization, astrogliosis was mitigated in the CNS—but not completely eliminated—suggesting that quiescent astrocytes can still become reactive even without Cav1.2 channels. Future experiments are needed to investigate the involvement of persisting Ca^++^-signaling pathways and the genes they regulate post-Cav1.2 channel deletion. RNA-Seq analysis on astrocytes isolated from dissociated spinal cords at various stages of EAE can potentially identify upregulated genes involved in astrocyte activation as well as transcription factors known to be activated by Cav1.2 channels. These experiments would provide crucial information about the molecular mechanisms by which Cav1.2 channels regulate astrocyte activation and may identify new transcription factors and intracellular pathways implicated in astrogliosis.

### Nonspecific blocking of Cav1.2 channels during EAE recapitulates conditional knockout findings

Voltage-gated Ca^++^ channel blockers are commonly used on human patients to treat absence seizures and hypertension, but recent evidence indicates their therapeutic efficacy for other maladies, such as Parkinson’s disease and drug addiction [39]. Inhibitors preferentially targeting Cav1.2 channels, such as nimodipine, have therapeutic potential for treating neuroinflammatory diseases, including MS [8, 27]. Nimodipine appears highly efficient in attenuating microglia activation to prevent neurotoxicity [13] and down-regulating the expression of pro-inflammatory factors TNFα and IL1β in the brain [26]. Although these findings suggest that nimodipine may be an important pharmaceutical for treating MS, the role of these Cav1.2 channel blockers in demyelinating conditions precipitated by autoimmunity has not been well-established.

Depleting reactive astrocytes during the acute phase of EAE worsens clinical outcomes and neuroinflammation while reducing astrogliosis in the chronic stage has opposite outcomes [23, 32]. Therefore, we wanted to assess potential time-dependent effects of nimodipine treatment in EAE, administering nimodipine during the *onset* (D7–17) or *peak* phase (D14–24) of the disease. In the present study, nimodipine seems to provide a beneficial suppression of astrocyte reactivity regardless of disease stage. Findings from nimodipine-treated EAE mice closely resemble those from our astrocytic Cav1.2^KO^ experiments. We observed less severe clinical signs associated with improved rotarod performance in the EAE chronic phase. Nimodipine administration at the peak significantly reduced maximum and cumulative scores, whereas nimodipine treatment throughout the onset of symptoms decreased the summation of scores. These data also support work by Ingwersen et al. [15] who found that nimodipine administered in the early stages and peak phase of EAE reduced clinical severity during the chronic phase independently of mouse strain and type of EAE model. This indicates that targeting Cav1.2 channels during any phase prior to the chronic phase will improve disease conditions downstream.

Histological changes were consistent with those observed in the Cav1.2^KO^ brain and spinal cord. When nimodipine was administered during EAE, both astrogliosis and microglia reactivity were significantly reduced in the cerebellum. Nimodipine treatment also resulted in decreased demyelination in the lumbar spinal cord, as well as a greater survival of myelin-producing cells, which likely resulted from the robust mitigation of neuroinflammatory cells. Since microglia do not express functional Cav1.2 channels [27], and these results highly correspond with our Cav1.2^KO^ findings, both decreased neuroinflammation and demyelination can be attributed to VGCC antagonization in astrocytes, specifically. Cav1.2 channels are also expressed by immune cells that produce cytokines in pathological conditions, such as T helper 2 (T_H_2)-lymphocytes [3], which get recruited into the CNS by reactive astrocytes during EAE, although no peripheral immune involvement has been reported in nimodipine-treated EAE animals [15, 27]. This study is limited by not including a detailed characterization of the electrophysiological properties of astrocytic VGCCs during EAE. Ex-vivo patch clamp recordings may generate critical insights about the activity of these channels under autoimmune (and normal) conditions that could be used to develop novel treatments that modulate astrogliosis and promote myelin repair in MS.

## Conclusion

This study investigated the role of astrocytic Cav1.2 channels in autoimmune-induced CNS inflammation and demyelination using the EAE model of MS. Both transgenic deletion and pharmacological antagonization of these channels resulted in an attenuated clinical profile during the EAE chronic phase, as well as reductions in reactive astrocytes, activated microglia, infiltrating lymphocytes, and myelin degradation in the lumbar spinal cord 40 day post-immunization. Our data suggest that inhibiting Cav1.2 channels in the CNS of EAE mice limits neuroinflammation and facilitates myelin survival through a change in the density and functionality of reactive astroglia. These findings may illuminate the therapeutic potential for brain-permeant dihydropyridines to target CNS inflammation and myelin loss in autoimmune demyelinating diseases.

## Data Availability

The data sets used and/or analyzed during the current study are available from the corresponding author on reasonable request.
